# Global hearing health: future directions

**DOI:** 10.2471/BLT.18.209767

**Published:** 2018-03-01

**Authors:** Shelly Chadha, Alarcos Cieza, Etienne Krug

**Affiliations:** a Blindness Deafness Prevention, Disability and Rehabilitation Unit, Department for Management of Noncommunicable Diseases, Disability, Violence and Injury Prevention World Health Organization, 20 Avenue Appia, 1211 Geneva 27, Switzerland.; b Department for Management of Noncommunicable Diseases, Disability, Violence and Injury Prevention, World Health Organization, Geneva, Switzerland.

Hearing loss is a growing public health issue of global concern. Although more hearing loss could be prevented and most people who live with unaddressed hearing loss could benefit from cost–effective interventions, the subject is often ignored.

Many causes of hearing loss that could be prevented persist or are on the rise. These include ear infections, the use of ototoxic medicines and noise exposure.^1^ Exposure to loud sounds in recreational settings is particularly concerning. Over one billion young people face the risk of developing permanent hearing loss due to the way they listen to music; this risk can be avoided through better awareness and legislation.^2^ In 2015, unaddressed hearing loss had a global cost of 750 billion United Sates dollars (US$).^3^ This figure is expected to rise as the number of people with hearing loss rapidly increases across the world.^4^ Predictions suggest that unless action is taken, as many as 630 million people could have disabling hearing loss by 2030; this number may reach nearly 900 million by 2050.^5^

Despite the overwhelming predictions, neither policy-makers nor the public prioritize hearing loss. This may be because hearing loss is a non-life-threatening, invisible disability.^1^^,^^6^ Although strategies for hearing care have been implemented in some countries, mainly in high-income countries, resource allocation and services for hearing loss remain scarce.^7^

Acknowledging the growing importance of this issue, the World Health Assembly (WHA) adopted a resolution for the prevention of deafness and hearing loss in 2017.^8^ This resolution urges national governments to act to reduce hearing loss and its impact. The resolution also calls all stakeholders in this field to take action, including World Health Organization (WHO) Member States, professional groups, nongovernmental organizations, civil society, academicians, clinicians, the private sector and United Nations agencies.

WHO hopes that this resolution will have an impact in the global health arena. Well-planned strategies need to be implemented in all parts of the world, including low- and middle-income countries, where most people with hearing loss reside.^9^ WHO has drawn attention to hearing health through the World Hearing Day, the Make Listening Safe initiative, and the preparation of technical tools to support Member States in preventing hearing loss and reducing its impact. However, the change that the resolution calls for requires a strategic and coordinated global effort.

Wilson et al. highlighted the need for a global initiative that facilitates efforts to stem and eventually reduce the rising prevalence of hearing loss.^1^ Perhaps the first step in developing such coordinated action is to establish a global alliance where stakeholders can interact and contribute towards a common objective. There are several examples where global action coordinated by a multistakeholder alliance has been instrumental in raising awareness and promoting activities on public health issues.^10^^–^^12^ The areas of road safety, violence prevention and health systems have all benefitted from such alliances. The field of global hearing health would do well to reflect and learn from these experiences in its quest for greater visibility and resources.

An alliance would have a cohesive body of stakeholders whose main purpose is to undertake advocacy and develop networks that facilitate knowledge sharing and foster opportunities for collaboration. With strong leadership, a diverse membership and sound financial backing, such an alliance would be well positioned to drive a global initiative. This alliance and its initiative could provide the impetus for implementing the WHA resolution on the prevention of deafness and hearing loss.

Such collaborative and determined action would enable protection of the hearing for over the billion people who are at risk, as well as allowing those who experience hearing loss to achieve their potential through equitable access to the required services and rehabilitation.
